# Aftercare following fatal traumatic injuries, needs and questions: a level 1 trauma center study and scoping review

**DOI:** 10.1007/s00068-025-02895-7

**Published:** 2025-06-16

**Authors:** Nadia A. G. Hakkenbrak, Johan G. H. van den Brand, Sohaib Jaddi, Linda J. Schoonmade, Frank W. Bloemers

**Affiliations:** 1 Department of Trauma surgery, Amsterdam University Medical Center, Amsterdam, the Netherlands; 2 Department of Trauma surgery, Northwest Clinics, Alkmaar, The Netherlands; 3 Medical Library, Vrije Universiteit, Amsterdam, the Netherlands

**Keywords:** Aftercare, Bereavement support, Grief, Questionnaire, Review, Trauma surgery

## Abstract

**Purpose:**

Approximately 2,000 people die each year in the hospital due to accidental or inflicted traumatic injuries in the Netherlands. This has major emotional and socioeconomic consequences. Bereavement support is offered to prevent complicated grief, however, recommendations on adequate aftercare by the hospital are lacking.

**Methods:**

Patients with fatal traumatic injuries admitted to the Northwest Clinics, Alkmaar, or Amsterdam University Medical Center, VUMC, between January 1st 2021, and January 1st 2023, were assessed for eligibility (Injury Severity Score ≥ 16, in-hospital mortality). Their relatives were contacted, and a questionnaire was administered to evaluate their experiences with the aftercare provided by the hospital. In addition, a scoping review was performed to report on recommendations to improve aftercare.

**Results:**

A total of 1,131 articles were identified for the scoping review, of which 10 were selected for analysis (four questionnaires and six interview-based studies). The implementation of grief services by skilled professionals is recommended. The most frequently reported time between death and contact was 4–6 weeks, with contact conducted via telephone. During the study period, 110 patients met the inclusion criteria for the questionnaire. The median age of the deceased was 70 years (SD 20); 58% were male, with a median Injury Severity Score of 26 (range 16–75). Bereavement support was offered to 50% of the relatives, requested by 34%, and absent or lacking for 24%.

**Conclusion:**

Aftercare following traum-related in-hospital deaths remains inconsistent. Both the questionnaire and scoping review recommend structured aftercare. Aftercare, by telephone or face-to-face, conducted by a trained professional four weeks after the death, is suggested to favorably influence the course of bereavement or lead to timely referral for grief counseling.

**Supplementary Information:**

The online version contains supplementary material available at 10.1007/s00068-025-02895-7.

## Introduction

Worldwide, fatalities due to trauma account for 4.4. million deaths every year, nearly 8% of all deaths [1].

In the European Union, 164,039 deaths result from trauma and constitute 3.1% of all deaths among residents [2]. In the Netherlands, the number of fatal accidents is even higher, with 7,290 traumatic deaths in 2021, accounting for 4.3% of all deaths [2]. Of these, approximately 2,000 people die each year in the hospital due to traumatic injuries; 900 of them die due to injuries with an Injury Severity Score (ISS) of 16 or higher [3].

The death of a loved one has a major impact, both emotionally and socioeconomically. Although the majority of bereaved relatives adjust well to the death of a loved one, a minority suffer from mental distress and complicated grief [4]. Traumatic bereavement, defined by Raphael and Martinek in 1997 as “losing a significant other due to sudden, violent, or accidental means”, may lead to posttraumatic stress, a disturbed grieving process and post-loss functional impairment [5–7]. In 10–20% of bereaved relatives, persistent psychiatric difficulties remain in the absence of grief interventions [8]. The impact on mental health varies, from separation distress to suicide, depending on the severity of grief symptoms.

Nowadays, bereavement support is offered to help relatives cope with the death of a loved one. Relatives are involved in end-of-life decision making in the hospital and supported throughout the hospital admission; aftercare is provided by the treating physician [9, 10]. Aftercare has also been described in the literature for relatives bereaved by suicide or combat [11, 12]. Aftercare is considered care focused on recovery or management of the grief process, e.g. referral to a bereavement or aftercare program. However, in most studies, bereavement support and aftercare programs focus on the support of bereaved caregivers after the loss of a child or long-term ill relative [13–16]. Due to its enormous emotional impact, the bereavement support program is described in detail for the pediatric population and end-of-life care [13–16].

Although the literature is limited and mainly focuses on pediatric and end-of-life care, trauma remains the leading cause of death in the population below the age of 50 years in the Western world [17]. Recommendations on how to provide adequate aftercare for relatives of patients who experienced traumatic fatalities are lacking, as there are no specifies studies on traumatic fatalities. Therefore, the aim of this study is to evaluate the experience of relatives bereaved by traumatic fatalities through a scoping review and questionnaire, from two major trauma centers in the Netherlands.

## Methods

A scoping review and questionnaire were performed to determine adequate aftercare provided by the hospital for relatives bereaved by traumatic fatalities. These will be addressed separately.

## Part I– scoping review

### Literature search

The primary aim of the scoping review is to identify what is considered adequate aftercare in the literature and to form recommendations on how to implement improvements in daily practice. This scoping review was conducted in accordance with the Preferred Reporting Items for Systematic Reviews and Meta-Analysis; Extension for Scoping Reviews statement (PRISMAScR) and registered at the Open Science Framework (10.17605/OSF.IO/SMHD8) [18]. A comprehensive search to identify studies on aftercare (provided by the hospital) was performed in the bibliographic databases Medline/Ovid, Embase.com, PsycInfo/Ebsco and Web of Science Core Collection/Clarivate, from inception to February 16, 2024, in collaboration with a medical librarian (LS). Search terms included controlled terms (MeSH in Medline, Emtree in Embase and Thesaurus terms in PsycInfo) as well as free text terms. The following terms were used (including synonyms and closely related words) as index terms or free-text words: ‘trauma’ and ‘death’ and ‘aftercare’ and ‘relatives’. The search was performed without any date or language restriction. Duplicate articles were excluded by a medical information specialist (LS) using DedupEndnote following manual deduplication in Endnote X20.5 (Clarivate^tm^) [19]. The full search strategy for all databases can be found in the Supplementary Information (Supplement 1).

### Selection process

The search findings were independently screened for eligibility by two authors (NH and SJ) using an online software program to facilitate the screening process (Rayyan, [20]). It was expected that the studies would consist of a very heterogenous and small population; therefore, inclusion criteria were broad. Studies were included if they met the following criteria: traumatic fatalities, age ≥ 18 years, aftercare, relatives, and availability of full-text articles. The exclusion criteria were children (< 18 years), non-traumatic fatalities, non-peer reviewed journals, books, theses, conference abstracts, editorials, and reviews. Differences in judgment between the two authors were resolved through a consensus procedure, and in cases of disagreement, a third author (FB) was involved.

## Quality assessment

The quality of the studies was assessed using the JBI Critical Appraisal Checklist for Qualitative Research and NIH Quality Assessment Tool for Observational Cohort and Cross-Sectional Studies [21–23]. The JBI checklist consists of 10 questions; if the answer is ‘No’ or ‘Unclear’, there is a risk of bias: selection bias (question 1,4,5), bias in measurement of outcomes (questions 2 and 3), bias in reported results (questions 6 and 7), and bias due to missing data (question 8) [24]. The NIH tool can award a maximum of 14 points for selection, comparability, and outcome. A score of 11 points or above was considered a good level of evidence, six points as fair, and five points or less as poor [21].

## Results

**Fig. 1:**
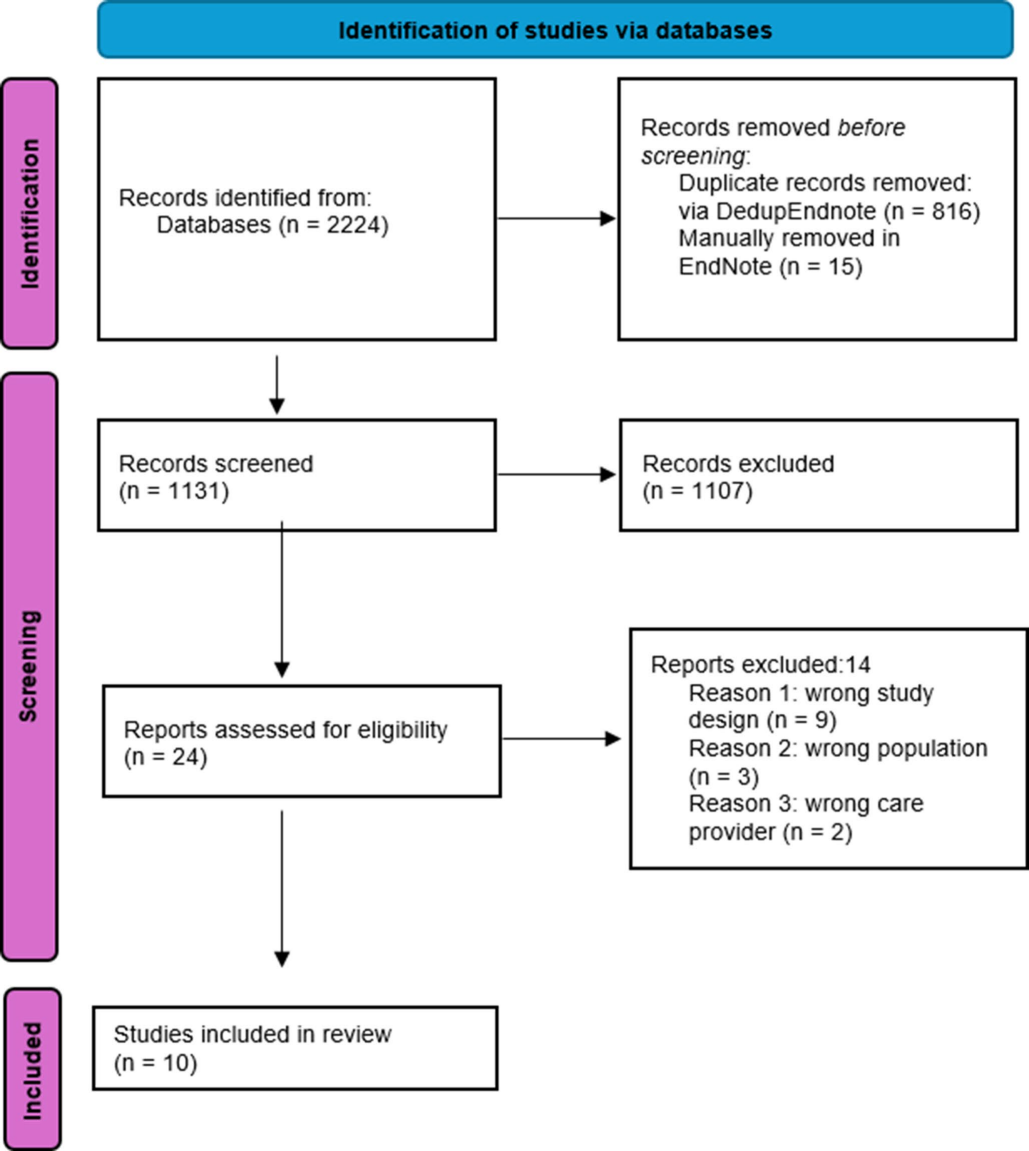
Flowchart of the search and selection procedure of studies ** PRISMA 2020 flow diagram template (PRISMA 2020 flow diagram — PRISMA statement (prisma-statement.org)

The literature search generated a total of 1,962 references: 409 in Medline, 433 in Embase, 344 in PsycInfo and 776 in Web of Science. After removing duplicates 1,131 references remained. The titles and abstracts of these articles were screened for eligibility, resulting in 24 articles for full-text screening. Ten articles met the inclusion criteria after full text screening (Fig. 1). The articles were assessed based on the following topics: participants, intervention, and recommendations. The studies were graded according to the JBI and NIH quality assessment tools (Table 1).

**Table 1:** Scoping review bereavement support and aftercare; A. Participants, B. Intervention and recommendations

Article	Country	Intervention	Fatality	Participants	Relationship	Mean age participants (Range)	Gender participants
1. Berry et al. 2013	United States	Questionnaire evaluating grief counselors experience in medical examiner's offices on the grief services program	Homicide (29%) Suicide (26%)Natural causes (20%)Accident (14%)	1085 contacts: - 28,4% first timer - 71,6% follow-up	Immediate family (91%)Extended family (5%) Friends (2%)Community groups (1%)	-	Male/female ratio 1.73
2. Buchanan et al. 1996	United States	Evaluating hospital trauma bereavement program	Trauma	347 families	Direct family members	-	-
3. Dyregrov et al. 2014	Norway	Questionnaire and interview with bereaved family members	Suicide (42%) Accident (42%) Sudden death child (11%) Homicide (5%)	30 family members (19 deaths)	Lost a child (60%) Lost a sibling (17%) Lost a parent (10%) Lost a niece/nephew (13%)	Mean age 46 years (35-55)	Female (67%) Male (33%)
4. Johnson et al. 2023	Norway	Questionnaire among close friends	Terrorist (bomb) attack	89 persons (67 deaths)	Close friends	Mean age 20.8 years (15-41)	Female (76%) Male (24%)
5. Katz et al. 2020	Australia	Questionnaire among relatives before and after patient’s death Electronic medical chart review of the patients	Trauma (motor vehicle collision and suicide)	Family members of the 159 patients who died within 72h of arrival (27 traumatic fatalities)	Family members	Mean age of the deceased 81 years (18-100)	-
6. Mitchell et al. 2016	Australia and New Zealand	Questionnaire among senior ICU nurses (adult, pediatric and neonatal) to describe the provision of family bereavement and provided support	Death in the ICU (including trauma)	153 participants (67% response rate):Australia: adult ICU nurses 63.3%New-Zealand: Mixed adult and pediatric: 52%	ICU nurses or managers	-	-
7. Parris et al. 2007	United Kingdom	Evaluation of the emergency department follow up by relatives, retrospective database audit	Death in the ER (including trauma)	478 relatives replied (response rate 41%): - 14% accepted the offer of a meeting (81% in department, 16% by phone and 4% by letter) -27% declined but almost all expressed gratitude for the offer	Spouses (54%) Child (25%) Sibling (9%) Parent (8%) Other (5%) Not reported (8%)	Age of the deceased: 41-60(24%) 61-80(50%) (Range 0-100)	-
8. Peters et al. 2016	Australia	In depth interview with the relatives on support	Suicide	10 Participants	Lost a spouse (50%) Lost a son (40%) Lost an uncle (10%)	-	Female (70%) Male (10%)
9. Rynearson et al. 1995	United States	Interview with relatives on demographic and risk factors, and self-report on trauma, dissociation and bereavement	Homicide	52 bereaved families (response rate 22%): 32 received additional treatment, 20 did not	Bereaved family members	-	-
10. Lloyd-Williams et al. 2009	United Kingdom	Interview with the relatives of deceased brain death ICU patients on death and donation	Brain death in the ICU (including traumatic brain injury)	29 relatives (response rate 22%)	Spouses 69% Parents 21% Children 7% Aunt 3%	Mean age participants 47years (SD 9.8) Mean age deceased 44 years (SD 15.2y)	Female (72%) Male (28%)
Article	Caregiver	Contact	Session	Time interval (death-contact)	Reason for contact	Recommendation/conclusion	NIH/ JBI (risk of bias)
1. Berry et al. 2013	-Clinical social workers (3) -On-call bilingual counselor (1) -Social work intern (1)	Physical visit (60,5%*) Telephone session (23.1%) Off-site visits (15.7%**) E-mail (0.7%) *one-on-one session (78.6%) ** (home visits (61.8%), support groups (38.2%)	Length: 30-59 minutes (36%)30.4% were 1-2hours (30.4%)15– 29 minutes (12.2%) < 15 minutes (17.0%)	0-1 year	-Counseling (74.1%).-Obtaining information (12.0%).-Autopsy results (6.6%)-Viewing of the body or photographs (3.4%)-Release of the decedent to the family for burial (2.1%)	- Implement grief services in ME/C offices to support individuals dealing with traumatic losses.- Ensure on-site or readily available access to licensed professionals skilled in traumatic death and crisis intervention.- Expand the reach of grief services using technology (such as telephone or online counseling).	No/limited risk (clinical demographics)
2. Buchanan et al. 1996	- Social workers and trained volunteers - Nurses and physicians (medical questions) - Clergy (crisis)	Sympathy card (100%) Telephone contact (58.2%) Handwritten notes, when unsuccessful telephone contact (30.8%) Anniversary and holiday letters (167%)	Length: Phone call (minutes– hours)	- 1 week: sympathy card - 2-4 months: at least 2 phone calls - Holiday cards and letters (Thanksgiving and winter holidays) - Anniversary letter at 1 year post-mortem	- Talk about the patient - Questions, reassurance about maximal care effort - Ask to visit the deceased’s room (re-experience grief and begin understanding and resolution of the event)	- The initial phone call should assess the support system, convey staff involvement and guide them to relevant resources. - Families with support systems and grief assistance are more likely to grieve healthy and avoid pathological grieving. -The treatment program provides closure and assists the family in its recovery. -Dedicated team.	Poor
3. Dyregrov et al. 2014	- Researchers with clinical experience and knowledge of bereavement and Sámi culture (an anthropologist and a psychologist)	In depth interview	Mean length: 2hours (Range 1-4hours)	Meantime 3 years (Range 1.5-7years)	Psychosocial help: -Information -Funeral or inquest attendance -Family counseling -Sick leave, medical prescriptions - Grief groups -Psychotherapy	Recommendation: - Early assistance should begin with a crisis meeting (< 14 days), followed by ongoing support and follow-up contact as needed. - Most bereaved individuals prefer active outreach, where helpers initiate contact early and offer support. Conclusion: - Experience; very/helpful (33%), helpful to a certain degree (33%) and not very helpful (33%). - Professional help had been offered in an unsystematic way. -Support from family and friends is not enough. Public help was requested. -Requested help: out-reach help, initial help and help over time, competent and adapted help. - Barriers to professional help: extremeness of the experience, inadequate help systems and prevailing norms.	No/limited risk of bias (recall bias)
4. Johnson et al. 2023	-	Questionnaire among close friends, invited by approaching the family members and inviting 4-6 close friends		At 18, 28, 40 and 102 months At 18 months 70% requested help, 42% received help (professional help/emergency services)	-	- Bereaved friends experienced high levels of grief over an extend period of 8.5 years. The greatest decrease was found between 2.5 and 3.5 years after the loss. - Females reported a higher need for help and received more help over time compared to males. - Receiving help was not related to changes in grief levels. - Those who reported a need for help over time reported less decrease in grief symptoms. - The low percentage of friends who received help over time may reflect on acknowledgement, validation and recognition as a bereaved.	Fair
5. Katz et al. 2020	-Social worker -Palliative care -Pastoral care -Grief counselor	Social work/Pastoral care: physical or telephone support Palliative care team: a condolence card, brochure about grief and bereavement with contact details for support, or a phone call	-	-	Grief, bereavement or end-of life support was addressed	Recommendation: -Assessment for complicated grief should be repeated and recognized as a potential outcome of admission. - Identify individuals at risk for complicated grief. -Develop standardized recourses and approaches to improve grief support in acute care setting. Conclusion: - Support provide before death (42.1%) and after death (27%). - 47.8% did not receive grief support prior or after death.	Fair
6. Mitchell et al. 2016	-Social worker (Australia) -Pastoral care -ICU nurses -General practitioners (New-Zealand)	Questionnaire	-	-Australia: <1 week follow-up call, annual visit of the ICU -New-Zealand: 4-6 weeks follow-up call, 6 weeks ICU visit	-	Recommendations - Standardize bereavement support across ICUs to ensure consistent, comprehensive care for all bereaved families.- Inform families about community bereavement services to support grieving, detect early signs of complicated grief, and reduce mental health risks.- Evaluate complex bereavement interventions from outcome and process perspectives to guide clinicians and policymakers on their effectiveness. Conclusion: - Viewing of the deceased in the ICU was offered in 96.6%. - Giving information to families about community bereavement services and sending a sympathy card: Australia (20.8%), New Zealand (54.2%). - Bereavement follow-up service was offered (31.9%). - Debriefing for staff after trauma (87.9%). - Follow-up offered by donate life staff (39.5%), social worker (67.4%) or other (55.8%). -Follow-up offered: call (80.9%), visit to the ICU (53.3%), formal counseling (64.6%), meeting with medical staff (91.4%).	Potential risk of bias (selection bias, information bias, confounding)
7. Parris et al. 2007	Bereavement consultant	- Invitation letter to plan a meeting	Length: 40-60 minutes (range 15min - 2h), single relative in 63% of the meetings	4-6 weeks after the loss	- Information about support (voluntary organizations, consultant) (81%) -Explanation of medical terms postmortem (75%) -Miscellaneous (17%) -Explanation of the circumstances per mortem (14%) - Medical screening of relatives (7%)- Reassurance that relatives could not have prevented death (6%)- Information about end-of life treatment (5%) - Medical follow-up, e.g. familial screening (13%)	- Reach out to relatives a few weeks after a death to answer forgotten questions and clarify facts, reducing guilt and aiding in the healing process by empowering them and preventing prolonged grief. - It is important to distinguish this follow-up service from a counselling service.	Risk of bias (selection, response, information, observer bias)
8. Peters et al. 2016	-Emergency workers -Coronial services -Health and Community Services -Peer support groups	In depth interviews	60-100 minutes	>12months	Themes discussed: -Seeking support from other bereaved -Initiating support -Facing insensitivity and experiencing compassion	- Interaction between the bereaved and care providers in the critical period following a suicide can favorably or adversely influence the course of bereavement. - Self-initiating support was experienced as challenging by the participant. Active intervention may prevent longer-term issues associated with complicated grief. - Peer support groups are highly valued for providing understanding from those with similar experiences.- Mental health nurses should educate first responders on the needs of those bereaved by suicide through their experience in mental health care.- Early intervention provides timely support to the bereaved, potentially easing acute grief symptoms.- Policy reforms in finance and social services are necessary to alleviate financial hardships that exacerbate the grieving process for those affected by suicide.	Limited risk of bias (recall, confirmation bias)
9. Rynearson et al. 1995	Psychotherapist (psychiatrist or psychologist)	Meeting (in-person)	Initial visit: 60 minutes	<3 months	-	Recommendation: - Reducing the impact of trauma must be the goal of treatment. While acknowledgement of and adjustment to the loss is a fundamental focus of treatment. - Risk factors for those who seek treatment are younger age and marital instability. Whilst affirmation of spirituality, religion or both are protective. - Increase in frequency of re-enactment imagery in unrecovered subjects. Conclusion: - Childhood history of sexual abuse and lack of religious faith were associated with treatment seeking. - Treatment-seekers scored higher on all measures of grief, trauma, and intrusive re-enactment imagery of the dying.	Potential risk of bias (selection bias)
10. Lloyd-Williams et al. 2009	Member of the supportive care group	Interview	-	-	Discuss brain death and donation at the ICU.	Recommendation: - Implement staff training on delivering bad news effectively.- Provide specialized end-of-life and bereavement support from a palliative care team.- Ensure continuous communication, possibly by involving families in ICU rounds.- Support families before, during, and after a loved one’s death with a multidisciplinary team. Conclusion: -Breaking bad news according to the well-recognized principles: first eliciting what the relatives may already know, establish how much information they want to know, and sensitively share information to their own pace and ability, constantly checking al information has been understood. - Lack of privacy at the ICU. - Contact with the medical team is appreciated, however, relatives find it difficult to initiate this contact themselves. - Donation agreement (75.8%).	Limited risk of bias (selection, recall bias)

### Participants

Of the included articles, three studies reported on suicide, three on homicide, and six did not specify the type of trauma. In four studies, a questionnaire was sent and in six studies the participating relatives were invited for an (in-depth) interview (Table 1).

The relationship of the relative to the deceased is outlined in Table 1: eight studies included relatives, one included close friends, and another included intensive care nurses and managers. The majority of participants were female, and the mean age ranged from 20.8 years to 47 years (Table 1).

### Intervention

Six studies reported the length of the interview sessions: five studies reported a length of approximately one hour, and one study reported phone calls lasting from a few minutes to several hours [25–30].

In four studies, the caregiver was a social worker, in two a bereavement consultant, in one a psychotherapist, and in two a member of the supportive care team (Table 1). In three studies, a physician or nurse was involved [28, 30, 31]. The time interval between the death and the moment of contact was described in nine studies, ranging from 4 to 6 weeks (by phone) to up to one year (Table 1).

### Recommendations to improve aftercare

Support should be provided during the critical period after the loss of a loved one, ideally within two weeks post-mortem [26, 28]. Interaction with the caregiver can favorably or adversely influence the course of bereavement. However, reaching out for help was experienced as challenging by relatives [28, 32]. As a result, several studies recommend implementing bereavement services led by a dedicated team of trained professionals [25, 30–33]. As active intervention may prevent complicated grief, it is also recommended to inform families about available bereavement support programs [26–28, 31]. Acknowledgement, validation, and recognition of bereaved relatives were found to be important [34]. Peer support groups were highly valued for offering understanding to those with similar experiences [28].

Risk factors for seeking treatment included younger age and marital instability, while spirituality and religion may be protective [29]. Females reported a higher need for help and received more help than males [31]. However, help received was not related to changes in grief levels, those who reported a higher need for help over time reported less of a decrease in grief symptoms [29, 34].

Two studies reported on the provided bereavement support: 76 relatives (47.8%) did not receive support prior to or after the death of their loved one, and bereavement follow-up services were offered to 46 relatives (31.9%) [31, 33].

## Part II– questionnaire

### Methods

The primary aim of the questionnaire was to evaluate aftercare provided at the two participating centers and assess the needs of bereaved relatives. Currently, aftercare consists of an interview conducted by the threating physician (trauma surgeon, intensive care physician, or emergency care physician) within two weeks up to three months post-mortem. However, there is no standard of care on the form, frequency, assigned physician and moment of follow up post-mortem. It is hypothesized that this current form of aftercare is insufficient and requires standardization.

### Questionnaire

All trauma patients with fatal traumatic injuries admitted to the Northwest Clinics Alkmaar or Amsterdam University Medical Center, VUMC between January 1 st 2021 and January 1 st 2023, were assessed for eligibility. Patients above the age of 18 years, with an ISS of 16 or higher, who died in the hospital, were included. The relatives registered as primary contacts of the deceased patient in the electronic patient registration system were located, and contact details were obtained to invite them to participate. Personal information from the relatives was not collected to maintain the anonymous and minimally invasive nature of the questionnaire. Data on age, gender, mechanism of trauma, and time to death were extracted from the Dutch national trauma registry (Landelijke Traumaregistratie, Netwerk Acute Zorg Noord-Holland/Flevoland).

A questionnaire was developed by the authors based on the results of the scoping review and clinical ambiguities.

Hereafter, eight questions were formed to evaluate the provided care, assess the desired form of aftercare, and identify any discrepancies. The questionnaire was reviewed by the department of communication of the participating centers (Supplement 2). Psychometric validation of the questionnaire was performed, focusing on adaptation and validation, until the research team was content [34, 35]. The study received approval from the institutional review board (2022.0718). The following questions were presented:

1. Did you experience sufficient support after the passing of your loved one?
2. Did you need care from the hospital after the passing of your loved one?
3. What form of post-mortem care did you receive?

What form of post-mortem care did you wish you had received?

4) When did you receive this form of post-mortem care (weeks from death to the moment of support)?
5) What would have been the ideal timing for receiving post-mortem care after the passing of your loved one?
6) How often would you have liked to receive post-mortem care?
7) Were there people not involved in post-mortem care that you would have liked to have been involved?
8) Who would you have liked to provide this post-mortem care?

The questionnaire was sent by post, as assigned by the Medical Ethics Review Committee. If the postal address was missing, incorrect or if no response was received, one of the authors (NH) followed up by telephone. If the initial attempt was unsuccessful, it was repeated a second time. A reminder was sent via email or phone three weeks after consent if a response was still not received. In accordance with the quantitative nature of the study, the results are presented descriptively.

## Results

### Questionnaire

During the study period, 193 patients with fatal traumatic injuries were admitted to the participating centers, 110 met the inclusion criteria. Of those, 32 relatives wanted to participate, 16 declined, and 37 could not be reached, resulting in a 29% participation rate. Two relatives who received the questionnaire found it to be intrusive and declined to participate.

**Fig. 2:**
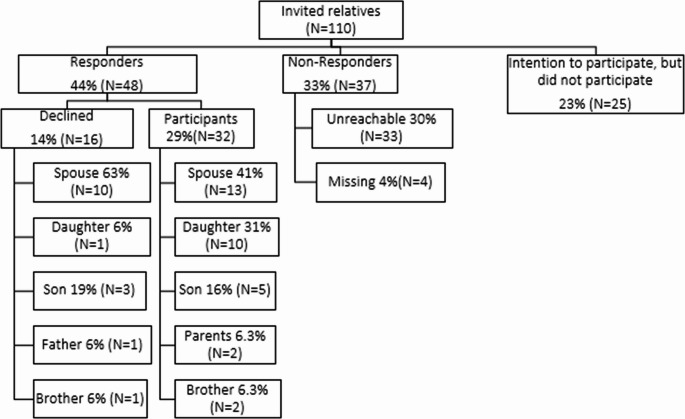
Response rate: 44%, 32 of the relatives participated and 16 declined

Overall, 58% of the patients were male, the mean age was 70 years (SD 20), and the median ISS was 26 (IQR 20–34). Up to 42% suffered fatal injuries due to a fall from height and 38% due to road traffic accidents. The median time to death was 2 days (IQR 0–6) (Table 2). In 13 fatalities the participating relative was a spouse and in 15 fatalities the son or daughter of the deceased (Fig. 2). The provided and preferred aftercare is described in Table 3 and presented below:

**Table 2:** Patient characteristics

Patient characteristics	*N* = 110 (%)
Gender	Male 64 (58%)
Age (mean, SD)	70 years (SD 20)
Mechanism of trauma:	
Fall from height	46 (42%)
Road traffic accident	42 (38%)
Stab- or shot incident	5 (5%)
Low energetic trauma	16(15%)
Injury Severity Score (ISS) (median, IQR)	26 (20-34)
Time until death (median, IQR)	2 days (0-6)

**Table 3:** Results Aftercare Questionnaire

Question		*N *= 32 (%)
Did you experience sufficient support after the passing of your loved one?	Yes, from my family and friends	24 (75%)
	Yes, from my family, friends and (medical) help	5 (16%)
	Yes, I sought or received (medical) help	1 (3%)
	No, my family and friends tried to help but it was not enough	1 (3%)
	No, namely…	1 (3%)
Did you need care from the hospital after the passing of your loved one?	Yes, I received it	3 (10%)
	Yes, but it was not offered	5 (17%)
	Yes, but it was not enough	2 (7%)
	No, but it was offered	10 (33%)
	No, it was not offered either	10 (33%)
What form of post-mortem care did you receive, or did you wish you had received?	In person	10 (46%)
	Telephone	8 (36%)
	Paper-based/email support	2 (9%)
	None	2 (9%)
What would have been the ideal timing for receiving post-mortem care after the passing of your loved one?	One week	5 (21%)
	One month	9 (37%)
	Six months	5 (21%)
	One year	5 (21%)
Where there people not involved in post-mortem care that you would have liked to have been involved?	Yes, namely the children	3(11%)
	Yes, namely the siblings	1 (4%)
	No	22 (82%)
	Other, namely	1 (4%)
Who would you have liked to provide this post-mortem care?	Grief counselor	4 (18%)
	Medical specialist (in training)	2 (9%)
	General practitioner	2 (9%)
	Intensive care physician	3 (14%)
	Other, namely	11 (50%)

#### Provided aftercare

- Aftercare provided by the hospital was reported as insufficient by 7 relatives (24%): not offered to 5(17%) and insufficient for 2 (7%). It was offered to 15 relatives (50%), requested by 10 (34%) and missed by 5 (17%).
- Thirty relatives (94%) reported sufficient support, mostly from family and friends (75%).
- Time to aftercare was reported in 14 cases, with a median of 4 weeks (IQR 1–7 weeks). Additional comments were: ‘one day after the passing away’, ‘first follow up after nine weeks and a second one after sixteen weeks’, and ‘after a few weeks, months.

#### Preferred aftercare

- The preferred form of aftercare was in-person for 10 relatives (46%) or by telephone for 8 (36%). Additional comments included: ‘I would have preferred support both by telephone and on paper’, ‘I received victim support’, and ‘I received support from the involved nurse, intensive care or general medicine physician’.
- The preferred timing of aftercare was diverse, one month (37%), one week, six months, and one year (21%).
- The favored frequency of aftercare was a single time for 12 relatives (55%) and weekly for 3 (14%). Additional comments were: ‘none (3x)’, ‘six months’, ‘six weeks’, ‘a few times with intervals of 6 months’, and ‘during a period of one year’.
- The preferred aftercare providers were grief counselors (18%), intensive care physicians (14%), medical specialists (9%), general practitioners (9%) and a combination of these (50%). Additional comments were: ‘both nurse practitioner and general practitioner’, ‘general practitioner and medical specialist’, ‘emergency care nurse’, ‘grief counselor and intensive care physician/general practitioner’, and ‘nurse practitioner and intensive care physician’.
- Requests to involve their children or siblings in the aftercare program were reported by 5 relatives (20%).

## Discussion

Traumatic bereavement, defined as the loss of a relative due to sudden, violent, or accidental means, may lead to a disturbed grieving process and functional impairment [5]. Complicated grief, a disturbed grieving process, characterized as intense and prolonged grief lasting more than 12 months in adults, is one such manifestation [11]. To ease the grieving process, bereavement support is offered in several ways and several intervention programs are developed [13, 14, 37].

The majority of bereavement studies include pediatric and palliative patients (e.g. cardiac, oncologic disease), with only a minority addressing adult trauma patients [13, 14, 37]. Since time to death is relatively short, compared to non-life threatening illnesses, a different form of aftercare and bereavement support may be required [8, 38].

In this study, hospital-provided aftercare was reported as insufficient by 7 relatives (24%). The majority of sufficient aftercare was provided by family and friends (75%). Even though this elucidates the importance of social support, it emphasizes the lack of structured, formal aftercare. McAdam et al. described an extensive bereavement support program that included an end-of-life brochure, a postcard at one week, follow-up at 4–5 weeks, six months, and one year [39]. However, the negative effect of postal cards and comprehensive programs have also been reported [40, 41]. Both the review and questionnaire showed variation in time to, though 4–6 weeks was frequently noted. While, two studies recommended aftercare within two weeks, the questionnaire results favored 4 weeks after death as time to aftercare [26, 28].

The frequency of aftercare varied widely in the scoping review, e.g. Buchanan et al. described an extensive program in 1986 that is comparable to McAdam [30, 39]. However, in the questionnaire the preferred frequencies of aftercare were a single time for 12 relatives (55%) and weekly for 3 relatives for a designated period of time (14%). This may be due to cultural differences, external bereavement support programs, or variations in general practitioner involvement across regions. Informing relatives about bereavement support programs may help prevent complicated grief [26–28, 31]. However, recognizing and acknowledging bereaved relatives may be difficult, especially for close friends and second-degree family members [36]. To identify those at risk, ongoing assessment for signs of complicated grief is warranted [33]. While participants generally favored a single aftercare contact, a second follow-up should be available upon request by the relative or treating physician. The preferred forms of aftercare, in the questionnaire, were in-person or by phone (46% vs. 36%), consistent with findings from the literature [37, 38].

In the questionnaire, 18% of the relatives preferred aftercare provided solely by a grief counselor, and 50% favored a combination of a grief counselor and physician. Although literature on adult trauma-bereavement is limited, all studies included in the review, involved caregivers with a social degree (social worker, bereavement consultant, or psychotherapist) (Table 1). This reinforces the importance of a specialized caregiver [29, 33]. Several studies also recommended implementing grief services led by a trained, dedicated team, as reaching out for help was experienced as challenging by relatives [25, 28, 30–33].

The data revealed that women reported a higher need for help and received more help than man [34]. Other risk factors for seeking help include younger age and marital status [29]. Nevertheless, receiving more help was not related to changes in grief levels [29–33, 36]. Prior research shows that relatives at high risk of prolonged grief disorder, from the loss of a spouse or child, reported greater access to mental health care professional, yet, experienced more frequently a lack of support [8]. The moderate risk group, those who lost a spouse, visited community groups and accessed palliative care services [7, 8]. For this group, social support was associated with reduced severity of depressive and posttraumatic stress disorder symptoms [42]. These findings highlight the importance of differentiating aftercare support from (comprehensive) bereavement programs for moderate- and high-risk bereaved.

Some limitations should be noted. First, the literature on adult (in-hospital) traumatic fatalities is limited. Only ten studies were eligible for analysis, and not all studies reported the number of traumatic fatalities in their study population. Secondly, due to the character of the studies, time between death and the moment of care and evaluation– up to over a year– is relatively long to recall emotions accurately and was frequently mentioned as reason not to participate in the questionnaire, this may introduce selection and recall bias. Third, the participation rate was no more than 29%. Several reminders were sent by email or phone, despite these efforts the participation rate did not increase. The participation rate is low for questionnaires, but equal or even relatively high for bereavement studies [8, 43]. Nevertheless, this may have introduced selection bias.

Moreover, factors such as ethnicity, religion and spirituality were not addressed in this study. A strength of this study is the representative sample of relatives, most of whom were spouses or children or the deceased.

This study reports a lack of consistency in aftercare provided to bereaved relatives following traumatic fatalities. This inconsistency is evident in the form, frequency, and assigned caregivers. Although a minority actively requested aftercare, the offer was appreciated by the majority of the relatives. Based on these findings, and considering clinical feasibility, the following aftercare is suggested: outpatient contact at one month, preferably conducted by telephone or in-person upon request, provided by a dedicated professional, ideally a grief counselor, physician or nurse who was involved in the deceased’s care. Future research should prospectively evaluate the implementation of such a revised aftercare program for relatives bereaved by traumatic fatalities.

## Conclusion

Bereavement support and active intervention may prevent complicated grief. For the majority of the bereaved relatives help from family and friends was sufficient (75%). However, aftercare from the hospital was lacking in 24% of the cases due to inconsistencies in form and availability. Although more than half of the relatives do not need aftercare, the offer is appreciated. Therefore, aftercare in the form of follow-up by telephone or upon request in person by a skilled professional is recommended. Four weeks after death seems to be an appropriate time for intervention, and to refer those in need of grief counseling.

## Electronic supplementary material

Below is the link to the electronic supplementary material.

Supplementary Material 1

## Data Availability

No datasets were generated or analysed during the current study.

## Declarations

### Data access

Research data supporting this publication are available in the Supplementary and upon reasonable request.
