# Supplementary material for: Aftercare following fatal traumatic injuries, needs and questions: a level 1 trauma center study and scoping review
Source: Eur J Trauma Emerg Surg. 2025 Jun 16;51(1):229. doi: 10.1007/s00068-025-02895-7 (PMC12170696; doi:10.1007/s00068-025-02895-7)
Supplement: Supplementary file 1 — Supplementary Material 1 [file 68_2025_2895_MOESM1_ESM.docx]

**Supplementary information S1.** Literature search

### Medline (Ovid) - History and Search Details February 16, 2024

|  | **Ovid Medline All <Coverage:– 1946 to February 15, 2024>** |  |
| --- | --- | --- |
| **Set** | **Search** | **Results** |
| #1 | Exp Aftercare/ OR exp Hospice Care/ OR (follow-up* OR after-car* OR aftercar* OR support*).ti,ab,kf | 3,428,624 |
| #2 | Exp Family/ OR (relatives OR family OR families OR parent* OR caregiver* OR caretaker* OR bereav* OR grief).ti,ab,kf | 1,857,197 |
| #3 | ((trauma OR traumatic OR injur*) ADJ3 (fatal* OR mortal* OR death*)).ti,ab,kf | 30,012 |
| #4 | 1 and 2 and 3 | 409 |

### Embase.com - History and Search Details February 16, 2024

| **Embase.com <Coverage: 1947 - February 16, 2024>** | | |
| --- | --- | --- |
| **Search** | **Query** | **Results** |
| #1 | 'aftercare'/exp OR 'bereavement support'/exp OR (follow-up* OR after-car* OR aftercar* OR support*):ti,ab,kw | 5,154,274 |
| #2 | 'family'/exp OR 'grief'/exp OR 'bereavement'/exp OR (relatives OR family OR families OR parent* OR caregiver* OR caretaker* OR bereav* OR grief):ti,ab,kw | 2,428,591 |
| #3 | ((trauma OR traumatic OR injur*) NEAR/3 (fatal* OR mortal* OR death*)):ti,ab,kw | 38,582 |
| #4 | #1 AND #2 AND #3 | 629 |
| #5 | #4 NOT ('conference abstract'/it OR 'conference review'/it) | 433 |

### APA PsycInfo (Ebsco) - History and Search Details February 16, 2024

| **APA PsycInfo (Ebsco) < Coverage: 1800 – February 16, 2024>** | | |
| --- | --- | --- |
| **Set** | **Query** | **Results** |
| S1 | DE ("Posttreatment Followup" OR "Aftercare") OR TI (follow-up* OR after-car* OR aftercar* OR support*) OR AB (follow-up* OR after-car* OR aftercar* OR support*) OR KW (follow-up* OR after-car* OR aftercar* OR support*) | 941,821 |
| S2 | DE ("Family" OR "Parents" OR "Family Members" OR "Fathers" OR "Mothers" OR "Spouses" OR "Caregivers" OR "Bereavement" OR "Grief") OR TI (relatives OR family OR families OR parent* OR caregiver* OR caretaker* OR bereav* OR grief) OR AB (relatives OR family OR families OR parent* OR caregiver* OR caretaker* OR bereav* OR grief) OR KW (relatives OR family OR families OR parent* OR caregiver* OR caretaker* OR bereav* OR grief) | 906,557 |
| S3 | TI ((trauma OR traumatic OR injur*) N3 (fatal* OR mortal* OR death*)) OR AB((trauma OR traumatic OR injur*) N3 (fatal* OR mortal* OR death*)) OR KW((trauma OR traumatic OR injur*) N3 (fatal* OR mortal* OR death*)) | 5,672 |
| S4 | S1 AND S2 AND S3 | 344 |

### Web of Science Core Collection - History and Search Details February 16, 2024

| **Web of Science Core Collection (Clarivate)  < Coverage: SCI-EXPANDED) 1900 - ; (SSCI) 1956 -; (AHCI) 1975 - ; (ESCI) 2005 - present>** | | |
| --- | --- | --- |
| **Set** | **Search Query** | **Results** |
| #1 | TS = (follow-up* OR after-car* OR aftercar* OR support*) | 4,831,253 |
| #2 | TS = (relatives OR family OR families OR parent* OR caregiver* OR caretaker* OR bereav* OR grief) | 4,278,603 |
| #3 | TS = ((trauma OR traumatic OR injur*) NEAR/3 (fatal* OR mortal* OR death*)) | 40,212 |
| #4 | #1 AND #2 AND #3 | 776 |

**Supplement 2. Aftercare questionnaire.**

1) Did you experience sufficient support after the passing of your loved one?

☐ Yes, from my family and friends

☐ Yes, I sought or received (medical) help

☐ No, but I did not need it

☐ No, my family and friends tried to help but it was not enough

☐ No, namely…

Additional comments:

2) Did you need care after the passing of your loved one from the hospital?

☐ Yes, I received it

☐ Yes, but it was not offered

☐ Yes, but it was not enough

☐ No, but it was offered

☐ No, it was not offered either

Additional comments:

3) What form of post-mortem care did you receive, or did you wish you had received?

☐ Telephone

☐ Paper-based or email support

☐ In-person support

Additional comments:

4) When did you receive this form of post-mortem care? (Number of weeks from death to moment of support)

5) What would have been the ideal timing for receiving post-mortem care after the passing of your loved one?

☐ One week

☐ One month

☐ Six months

☐ One year

☐ Other, namely…

Additional comments:

6) How often would you have liked to receive post-mortem care?

☐ Once only

☐ Weekly, for a period of … weeks/months

☐ Monthly, for a period of … months

☐ Multiple times, with intervals of … weeks/months

☐ Other (please provide information in comments below)

Additional comments:

7) Where there people not involved in the post-mortem care whom you would have liked to have been involved?

☐ Yes, namely the children

☐ Yes, namely the siblings

☐ Yes, namely the parents

☐ No

☐ Other, namely…

Additional comments:

8) Who would you have liked to provide this post-mortem care?

☐ Nurse practitioner

☐ Professional specialized in grief counseling

☐ Medical specialist (in training)

☐ General practitioner

☐ Intensive care physician

☐ Other, namely

Additional comments:
